# Universal capillary screening for chronic autoimmune, metabolic and cardiovascular diseases: feasibility and acceptability of the UNISCREEN study

**DOI:** 10.3389/fpubh.2025.1506240

**Published:** 2025-02-11

**Authors:** Sara Angiulli, Aurora Merolla, Elisa Borgonovo, Rebecca De Lorenzo, Serena Spadoni, Barbara Fontana, Giuseppina Manganaro, Elena Rela, Alberto Bongiovanni, Rita Peracino, Chiara Bellino, Giulia Pata, Eleonora Bianconi, Sabina Martinenghi, Francesca Ulivi, Cristina Renzi, Emanuele Bosi, UNISCREEN Study Group, Sara Angiulli

**Affiliations:** ^1^Vita-Salute San Raffaele University, Milan, Italy; ^2^Diabetes Research Institute, IRCCS San Raffaele Hospital, Milan, Italy; ^3^Fondazione Italiana Diabete (FID), Milan, Italy; ^4^Behavioural Science and Health, Institute of Epidemiology & Health Care, University College London, London, United Kingdom

**Keywords:** feasibility and acceptability, population screening, capillary blood, type 1 diabetes, celiac disease, type 2 diabetes, dyslipidemia, cardiovascular diseases

## Abstract

**Background:**

UNISCREEN is a general population study aiming at evaluating feasibility and acceptability of universal screening for chronic autoimmune (type 1 diabetes and celiac disease) and metabolic/cardiovascular diseases (dysglycemia, type 2 diabetes, dyslipidemia, hypertension) across all age groups using capillary blood sampling by fingerprick to measure disease risk markers.

**Methods:**

UNISCREEN was conducted in the Cantalupo fraction, Cerro Maggiore, Milan, Italy, counting 3,061 inhabitants between 1 and 100 years of age. Participation was voluntary, following a public call. Participants were invited to respond to feasibility and acceptability questionnaires immediately before and after the screening procedures, which included the following: capillary blood drawing for immediate measurement of metabolic parameters (glucose, glycated hemoglobin, total-, HDL- and calculated LDL-cholesterol, triglycerides) and late autoantibody assays; blood pressure measurement; brief consultation with a physician providing feed-back on immediate results and health recommendations. The study was registered as ClinicalTrials.gov NCT05841719.

**Results:**

The study included 1,535 participants (50.1% of local population). A single fingerprick was sufficient for all measurements in 47.1% of cases, while up to two were necessary in 86.9% of cases. Complete glucose and lipid panels were obtained in 1382 participants (90.0%). Sufficient serum for late autoantibody testing was obtained in 99.7% of participants. The questionnaires showed overall satisfaction, with over 90% of participants considering capillary sampling simple and practical, and preferring it to venous sampling. Before screening, 24.0% adults and 31.7% children’s parents were worried about the possibility of being diagnosed or identified as being at risk; worries decreased to 21.3 and 23.4%, respectively, after medical consultation. The immediacy of some result communication possibly contributed to reducing the anticipatory anxiety.

**Conclusion:**

The UNISCREEN study shows that universal screening for chronic autoimmune, metabolic and cardiovascular diseases in the general population using capillary blood testing is feasible and acceptable.

## 1 Introduction

Universal screening for prediction or early identification of highly prevalent chronic autoimmune, metabolic and cardiovascular diseases in the general population might represent a pragmatic approach for reducing the public health burden of these diseases, consistent with the consolidated Wilson and Junger’s principles of screening (1).

The UNISCREEN study was conceived and designed as a population study, conducted in a small community in Northern Italy (2).

The aim of this study was to assess feasibility and acceptability of UNISCREEN, a population screening program across all ages. The study was conducted at a single center, based on voluntary participation, using capillary blood sampling for the measurements of validated markers of risk for type 1 diabetes, celiac disease, type 2 diabetes, dysglycemia and dyslipidemia, in addition to blood pressure measurements.

## 2 Materials and methods

### 2.1 Study design

The UNISCREEN study protocol has been recently reported in detail (2). The protocol and the standard informed consent forms (together with the anamnestic forms and questionnaires) were reviewed and approved by the San Raffaele Hospital Ethics Committee. The study was registered as ClinicalTrials.gov, NCT05841719.

Briefly, screening was offered to people aged 1 to 100 years, resident in the Cantalupo fraction of Cerro Maggiore municipality, Milan, Italy, who voluntarily participated in response to a public call. Adult participants signed an informed consent, while one of the parents signed for minors. All the procedures took place at the Municipal School in Cantalupo, carried out by a team of physicians and nurses from the San Raffaele University Hospital, with the collaboration of volunteers from Fondazione Italiana Diabete and several associations, including the local Civil Defence, Red Cross and Rotary Club. A brief medical interview was conducted by a physician, collecting baseline characteristics on demographics, anthropometrics (self-reported height and weight) and medical history, with a specific focus on diabetes mellitus, celiac and cardiovascular diseases. Blood pressure was then measured electronically (Omron M6, Omron Healthcare) by two volunteers from the Red Cross in sitting position after 5 min of rest, at dominant upper forearm. The measure was repeated twice, a few minutes apart, and both values of systolic and diastolic blood pressure were recorded.

Each participant underwent capillary blood sampling by one fingerprick, repeated in case of insufficient blood specimen. Samples were used for collection in microtubes (Microvette® 200 Z tubes) of at least 25 μL volume for future autoantibody assays and for extemporary measurements of glucose (0.6 μL) by glucometer (Accu Chek Inform II, Roche Diagnostics), HbA1C (6 μL) and lipids (total, HDL- and LDL-cholesterol and triglycerides, 19 μL) by point-of-care (POC) devices (Cobas b 101, Roche Diagnostics, for both HbA1c and lipid panel on separate diskettes). Sampling of venous blood was requested in case of capillary blood drawing failure.

The physician, during a brief consultation with participants, provided immediate feed-back and health recommendations both to participants and their general practitioner (GP) based on results of measured glucose, lipids and blood pressure, considering the baseline medical condition and current therapy.

Centrifuged serum samples from microtubes were obtained for autoantibody measurements and stored at −20°C until assay. Autoantibody measurement was performed by Luciferase Immuno Precipitation System (LIPS) and included the type 1 diabetes specific antibodies to glutamic acid decarboxylase (GAD), insulin, tyrosine phosphatase (IA-2) and zinc transporter 8 (ZnT8) (3–6) and celiac specific IgA and IgG antibodies to transglutaminase (7). Participants who tested positive to any autoantibody, were subsequently invited to perform a confirmatory test on venous sampling.

Feasibility and acceptability were assessed with questionnaires administered to each participant immediately before and immediately after the screening procedure. The questionnaires, rather than being self-completed by participants, were administered by a specifically trained non-medical member of the UNISCREEN team, in order to optimize the understanding of each question and reduce the risk of missing responses. For participants younger than 16, responses were provided by one or both parents. Pre-screening questionnaires included 11 questions, 8 on acceptability and 3 on feasibility. Post-screening questionnaires included 15 questions, 8 on acceptability (the same 8 as pre-screening) and 7 on feasibility (with one being the same as pre-screening). The same questionnaires were employed for adults and minors (Supplementary Table S1). Questions covering acceptability items (A to H of the ‘pre-screening’ and A’ to H’ of the ‘post-screening’ questionnaire) explored participants’ agreement with screening objectives, understanding of the screening plan, perceptions of the program’s safety and impact on their quality of life. Questions covering feasibility (I to K of the ‘pre-screening’ and I’ to Q of the ‘post-screening’ questionnaire) explored concerns about the capillary sampling procedure, feelings about the potential diagnosis of the screened conditions and the overall satisfaction about the program.

### 2.2 Outcome measures

Both feasibility and acceptability were measured using the questionnaires to collect data on participant’s evaluation of the screening plan and methods. Each question resulted in a single answer measured on a 5-point ordinal scale: strongly disagree (1 point); disagree (2 points); neutral (3 points); agree (4 points); and strongly agree (5 points).

Moreover, acceptability was also assessed by dropout rate, while feasibility was assessed by participant attendance: as pre-specified in the original protocol (2), for a successful recruitment the minimum necessary number of screened people was half of the total population plus one. The number of fingerpricks per participant, the need for venous sampling and any adverse events associated with the screening-procedure were recorded for each participant to evaluate feasibility. Sample collection was defined as successful when providing sufficient volume and quality to ensure reliable results for both metabolic and autoantibody tests.

### 2.3 Statistical methods

Descriptive statistics were performed for all variables, with continuous variables being expressed as medians and interquartile ranges (IQRs), while categorical variables as absolute counts and proportions (%). The Mann–Whitney *U* test and the χ2 tests were used to compare medians and proportions, respectively.

Response rates (i.e., the percentage distribution of enrolled participants out of the total number of inhabitants) were calculated for the overall population and according to age groups (1–15, 16–30, 31–45, 46–60, 61–75, 76–100 years).

The Wilcoxon Signed-Rank test for paired samples was used to compare questionnaire responses before and after screening. We compared overall answers for all participants, and also examined answers separately for participants aged >16 years and of parents of participants <16 years of age.

Statistical analyses were conducted by Stata18.0 (StataCorp. 2023. Stata Statistical Software: Release 18. College Station, TX: StataCorp LLC.), with a two-sided significance level set at *p* < 0.05.

Missing data are labelled with letter “a” (Supplementary Tables S2, S3).

## 3 Results

### 3.1 Study participation

From April 22, 2023, to October 29, 2023, 1,535 people, representing 50.1% of the 3,061 inhabitants of Cantalupo between the ages of 1 to 100 years, participated in the UNISCREEN study. Table 1 shows their baseline characteristics.

**Table 1:** Characteristics of screened population.

Baseline characteristics of participants (*N* = 1,535)	
Age, median (IQR)	49 (27, 64)
Age categories, n(%)	
0–15	242 (15.8%)
16–30	173 (11.3%)
31–45	283 (18.4%)
46–60	375 (24.4%)
61–75	323 (21.0%)
76–100	139 (9.1%)
Female, *n* (%)	845 (55.0%)
Smoking ^a^, *n* (%)	
Never	1,023 (67.9%)
Active	268 (17.8%)
Ex-smokers	216 (14.3%)
Family setting^a^, *n* (%)	
Alone	165 (11.0%)
With family	1,340 (89.0%)
Body Mass Index (BMI) ^a^, median (IQR)	23.3 (20.3, 26.4)
Lifestyle ^a^, *n* (%)	
Sedentary	601 (40.0%)
Active	900 (60.0%)
Pregnancy ^a^, *n* (%)	501 (59.3% of females)
Gestational diabetes ^a^, *n* (%)	26 (5.19% of pregnancies)
Diabetes Mellitus ^a^, *n* (%)	
Type 1	5 (0.3%)
Type 2	73 (4.8%)
Hypertension ^a^, *n* (%)	359 (23.8%)
Cardiovascular events ^a^, *n* (%)	67 (4.5%)
Active therapy for Diabetes Mellitus ^a^, *n* (%)	
Insulin	9 (0.6%)
Other medications	54 (3.6%)
Insulin and other medications	5 (0.3%)
Active therapy for hypertension ^a^, *n* (%)	332 (22.0%)
Active therapy for dyslipidaemia ^a^, *n* (%)	264 (17.5%)
Cardioaspirin ^a^, *n* (%)	102 (6.8%)
Oral contraceptives ^a^, *n* (%)	93 (11.0% of females)

Data are referred to the whole population. In people aged less than 16 y.o., questions were addressed to parents. Missing data are labelled with letter “a” (Supplementary Tables S2, S3). IQR, interquartile range.

**Figure 1:**
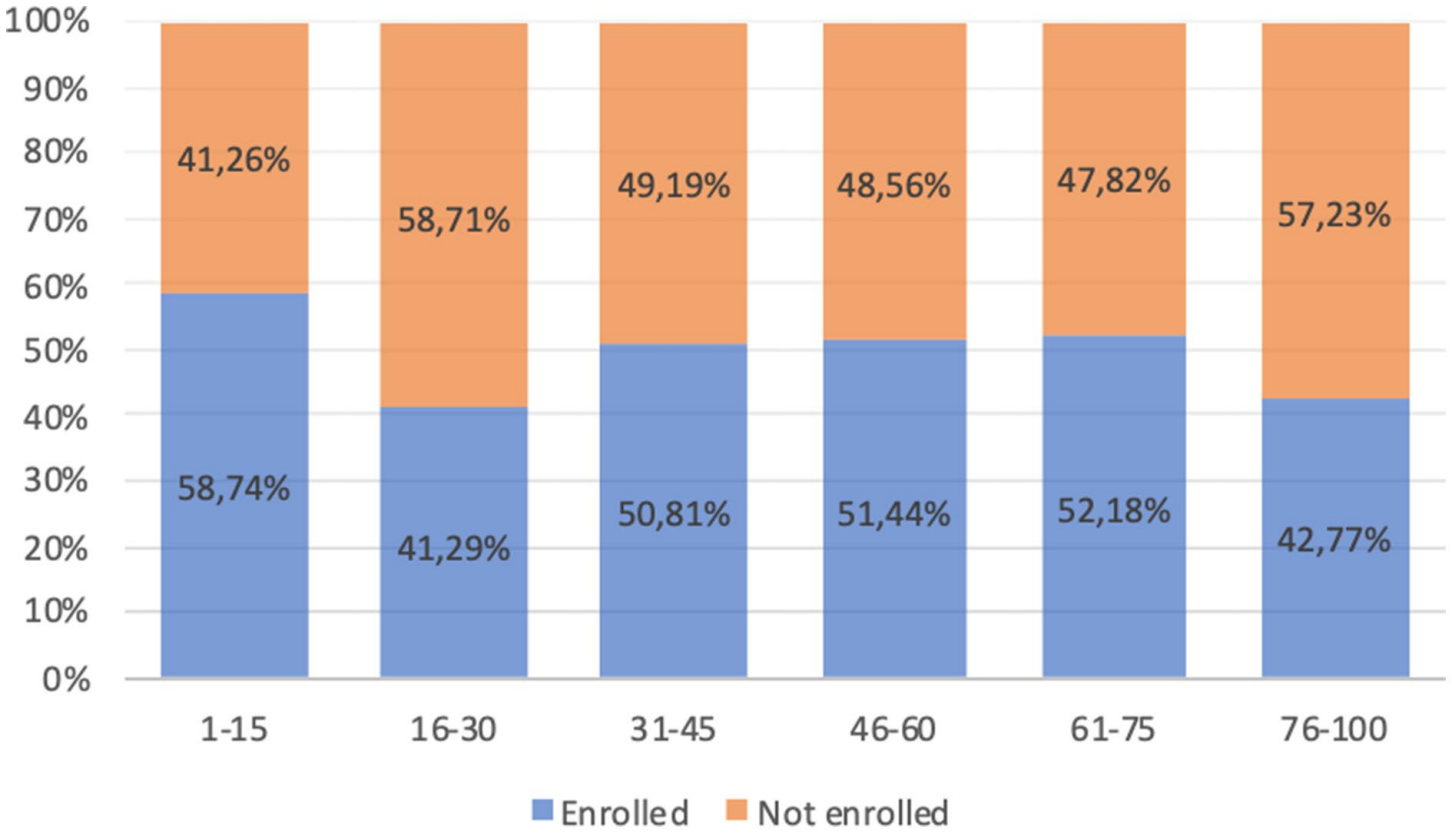
Response rates (%) in different age groups.

Response rates significantly differed across age groups (*p* < 0.001) as depicted in Figure 1, with two age groups, 16–30 and 76–100 years of age, being underrepresented. Additionally, females were more likely to be enrolled than men were (54.3% vs. 45.9%, *p* < 0.001, Supplementary Figure S1).

### 3.2 Successful samplings and measurements

The fingerprick procedure was able to provide sufficient capillary blood for sampling in microtubes, strips and diskettes in almost all participants, with only 5 (0.3%) requiring venous blood sampling. A single capillary sample was sufficient for all measurements in 47.1% of participants, whereas 39.8% required two, 10.2% required three, 2.0% required four and 0.9% required five of more fingerpricks. (Supplementary Figure S2). The need to perform more than 1 fingerprick was different across age categories (*p* = 0.030): participants in the 75–100 age range were more likely to receive more than one fingerprick (61.1%) compared to those in the 61–75 and 16–30 age group (50.6 and 43.9% respectively, Supplementary Figure S3). The need to perform more than 1 fingerprick was not associated with sex, smoking habit or the presence of diabetes.

Rates of successful glucose and lipid measurement by POC devices varied by the specific marker, with the highest success for blood glucose as shown in Table 2. Whole glucose and lipid panels were obtained in 1382 participants (90.0%).

**Table 2:** Measurements obtained with POC devices.

POC measurements (*N* = 1,535)	
Blood glucose, *n* (%)	1,529 (99.6%)
HbA1c, *n* (%)	1,515 (98.7%)
HDL-c, *n* (%)	1,460 (95.1%)
LDL-c, *n* (%)	1,389 (90.5%)
Total cholesterol, *n* (%)	1,472 (95.9%)
Tryglicerides, *n* (%)	1,466 (95.5%)

HbA1c, glycated hemoglobin. HDL-c, high-density lipoproteins cholesterol. LDL-c, low-density lipoproteins cholesterol. POC, point-of-care.

Sufficient serum for late autoantibody testing was obtained in 99.7% of participants.

At least one measurement of blood pressure was successfully recorded in 97.7% of participants (86.8% of children under 16 years of age and 99.7% of adults), while double measurement was obtained in 92.9% of participants (69.8% of children under 16 years of age and 97.2% of adults).

### 3.3 Feasibility and acceptability questionnaires

#### 3.3.1 Acceptability questions

**Figure 2:**
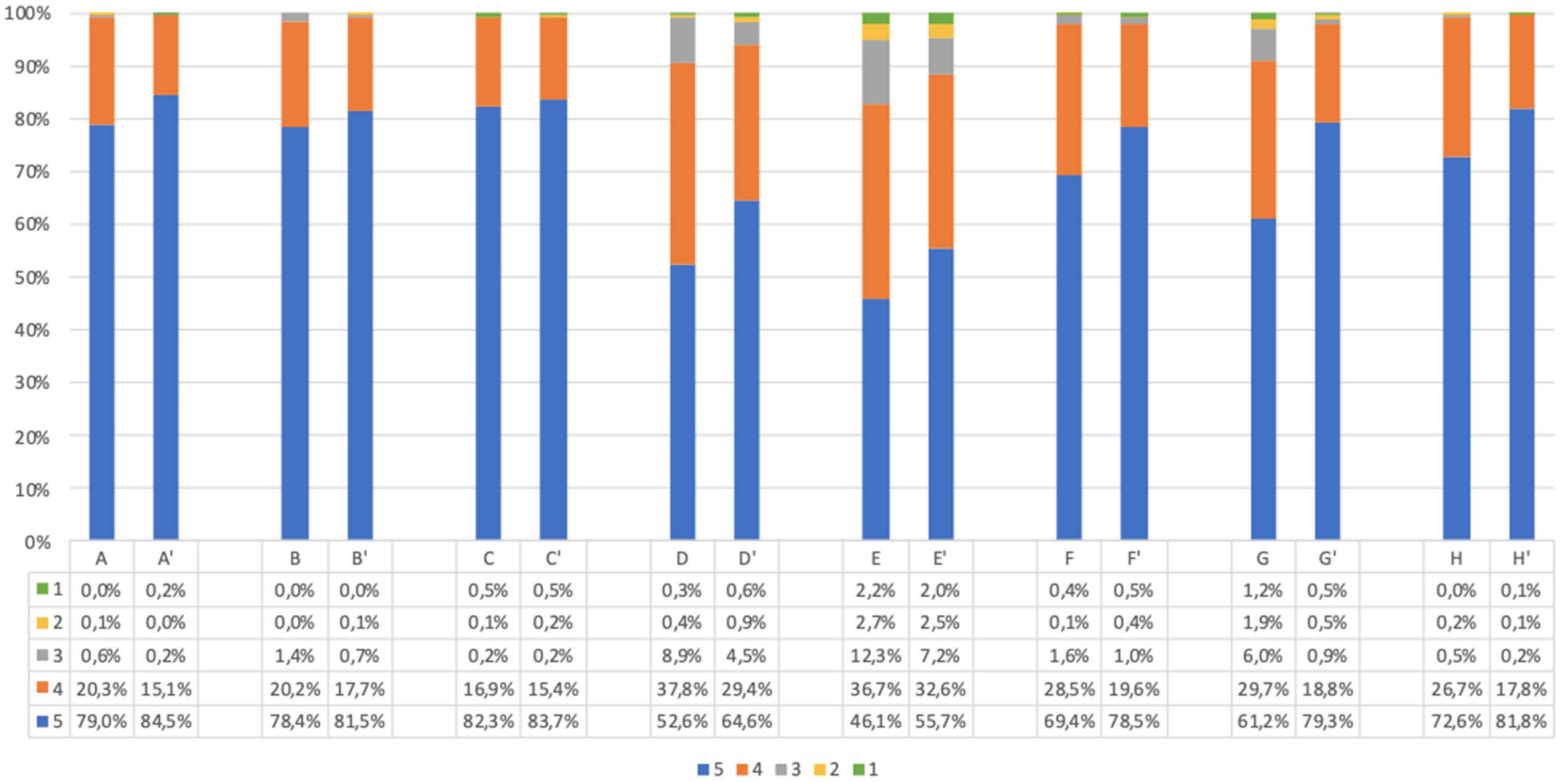
Adults questionnaire acceptability answers (%) immediately before and immediately after screening procedure. Answers were given on a 5-point ordinal scale: strongly disagree (1); disagree (2); neutral (3); agree (4); and strongly agree (5). (A,A’) I believe this screening program will be useful for general population to prevent future health problems. (B,B′) I believe this screening program will be useful for me to prevent future health problems. (C,C′) I support the idea of population screening for diabetes, celiac disease and cardiovascular disease. (D,D′) I believe this program will improve my quality of life. (E,E’) I believe this program will help me change my lifestyle. (F,F′) I find this program safe in terms of health effects and data confidentiality. (G,G’) I have understood what I would have to do in case of a positive screening test result. (H,H′) I understood the purpose of the program. Missing data are available in Supplementary Tables S2, S3.

**Figure 3:**
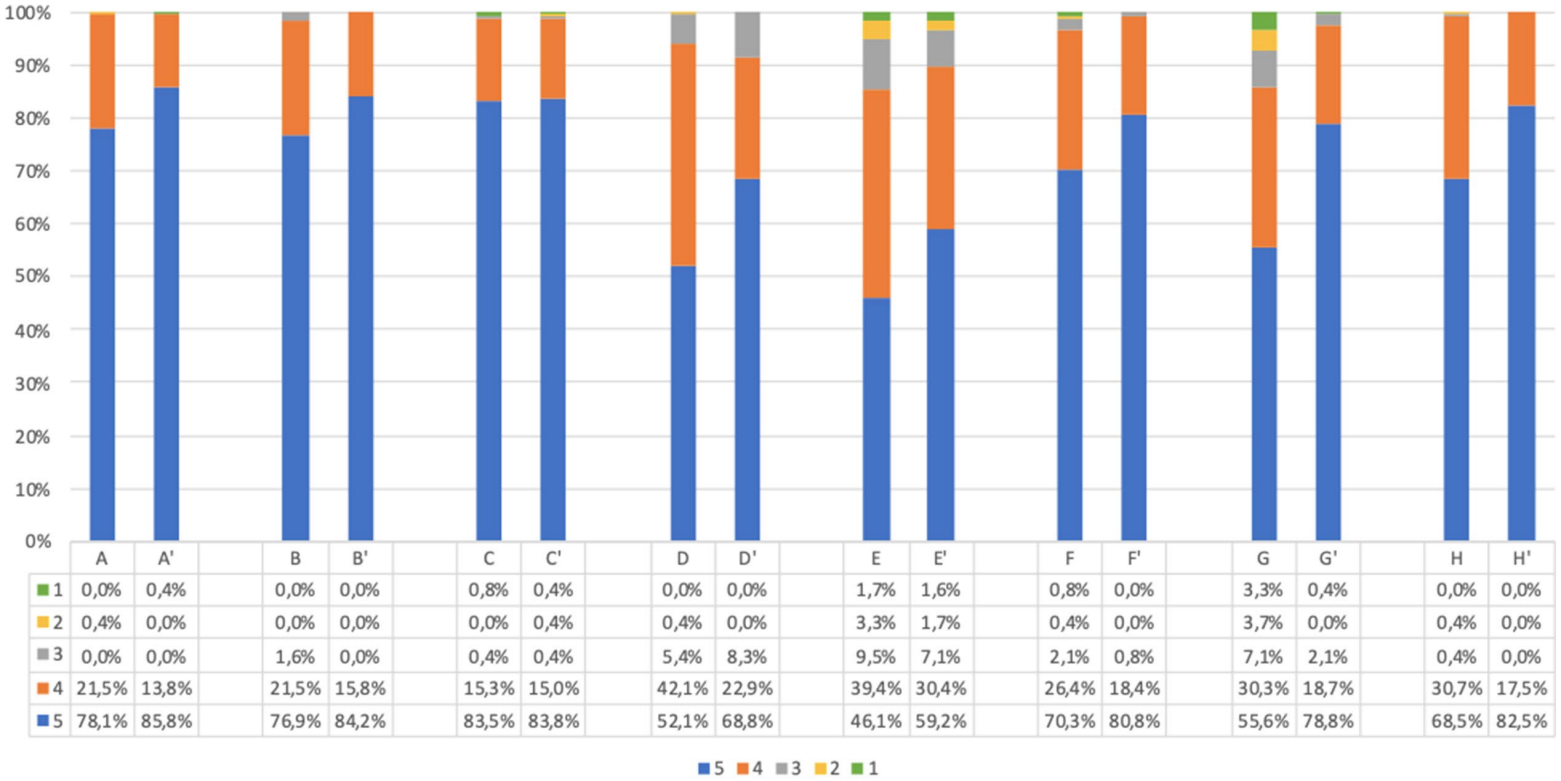
Children’s questionnaire acceptability answer (%) immediately before and immediately after screening procedure. Answers were given on a 5-point ordinal scale: strongly disagree (1); disagree (2); neutral (3); agree (4); and strongly agree (5). (A,A’) I believe this screening program will be useful for pediatric population to prevent future health problems. (B,B′) I believe this screening program will be useful for my child to prevent future health problems. (C,C′) I support the idea of population screening for diabetes, celiac disease and cardiovascular disease. (D,D′) I believe this program will improve my child’s quality of life. (E,E’) I believe this program will help my child change his/her lifestyle. (F,F′) I find this program safe in terms of health effects and data confidentiality. (G,G’) I have understood what my child would have to do in case of a positive screening test result. (H,H′) I understood the purpose of the program. Missing data are available in Supplementary Tables S2, S3.

The responses to acceptability questions in adults, before and after screening, are reported in Figure 2. The large majority of participants agreed on acceptability of the UNISCREEN study (considering the sum of point 5 (strongly agree) and point 4 (agree) items on the questionnaire). A large percentage of adult participants supported the idea of systematic screening for diabetes, celiac disease and cardiovascular diseases (99.1% after screening, question (C′)), with no significant difference in answers before and after screening (*p* = 0.486). The majority of adult participants believed that our program could be useful both for the general population (99.6% after screening, question (A’)) and for themselves (99.2% after screening, question (B′)) to improve their health status and prevent future health problems. After screening, 93.9% of adults believed that the program could improve their quality of life (question (D′)), while 88.8% believed the program could help them change their lifestyle (question (E’)). Almost all adult participants found the program safe (98.1% after screening, question (F′)) and understood its purpose and implications (99.7% after screening, question (H′)); the diagnostic process to be followed in the event of a positive test result was clear to the 98.1% of adults (question (G’)). The answers from children’s questionnaires, obtained from parents, were similar to those of adults (Figure 3).

The comparison of answers before and after screening showed that there is a statistically significant difference between the responses (*p* < 0.001), with the exception of question C-C′. In all answers, we observed an increase of positive responses after screening. The same trend was confirmed when considering separately adults and parents of children.

#### 3.3.2 Feasibility questions

**Figure 4:**
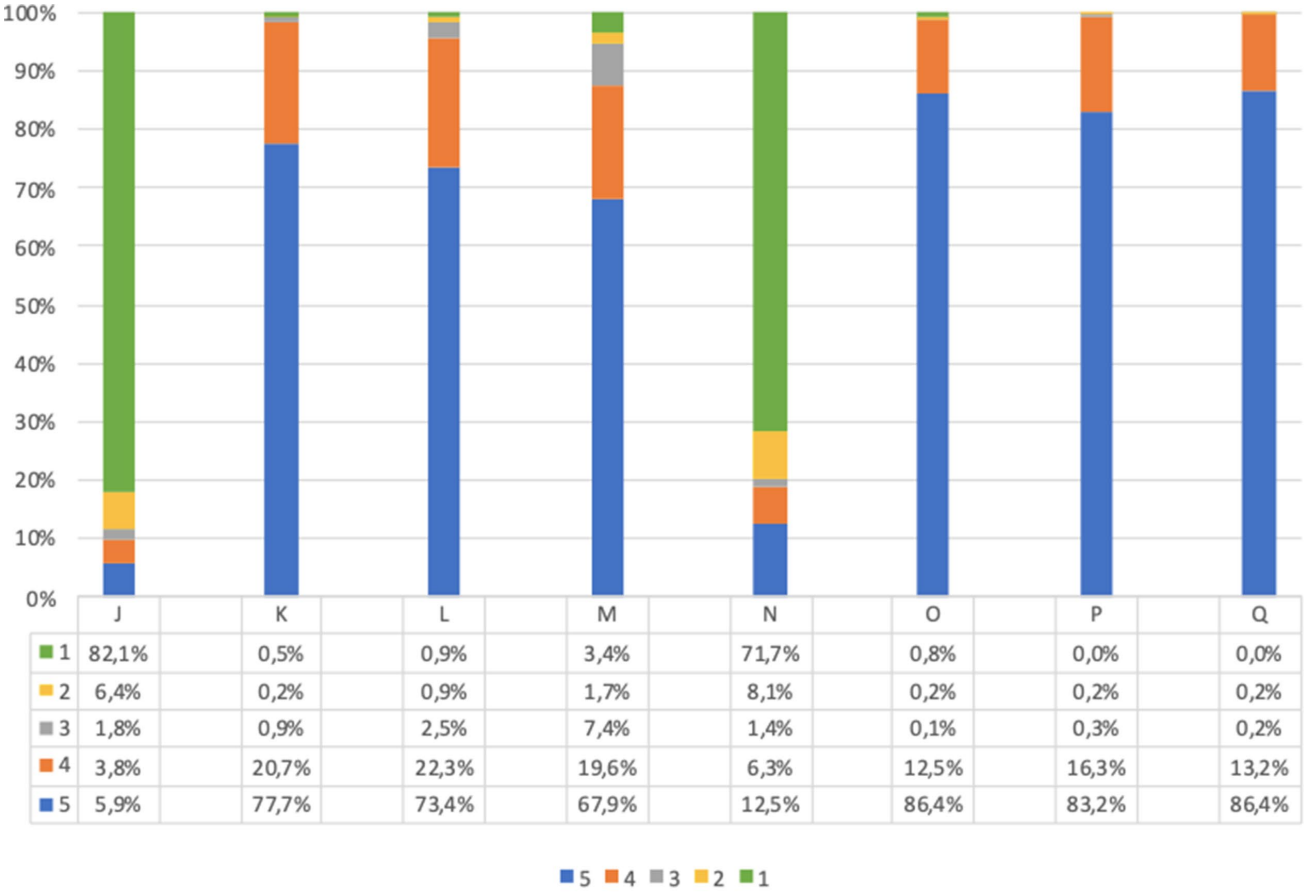
Adults questionnaire feasibility answers (%) immediately before and immediately after screening procedure. Answers were given on a 5-point ordinal scale: strongly disagree (1); disagree (2); neutral (3); agree (4); and strongly agree (5). (J) I am concerned about the idea of taking a capillary sample. (K) If I test positive for type 1 diabetes and/or celiac disease, I would agree to take a confirmatory venous blood sample, as required by the program. (L) I believe capillary screening is practical and easy. (M) I would prefer capillary sampling as a screening method over venous blood sampling. (N) What I expected from the program was different from what I got. (O) I am satisfied with the service I received. (P) All information about the program was clear and easy to understand. (Q) I would recommend this screening program. Missing data are available in Supplementary Tables S2, S3.

**Figure 5:**
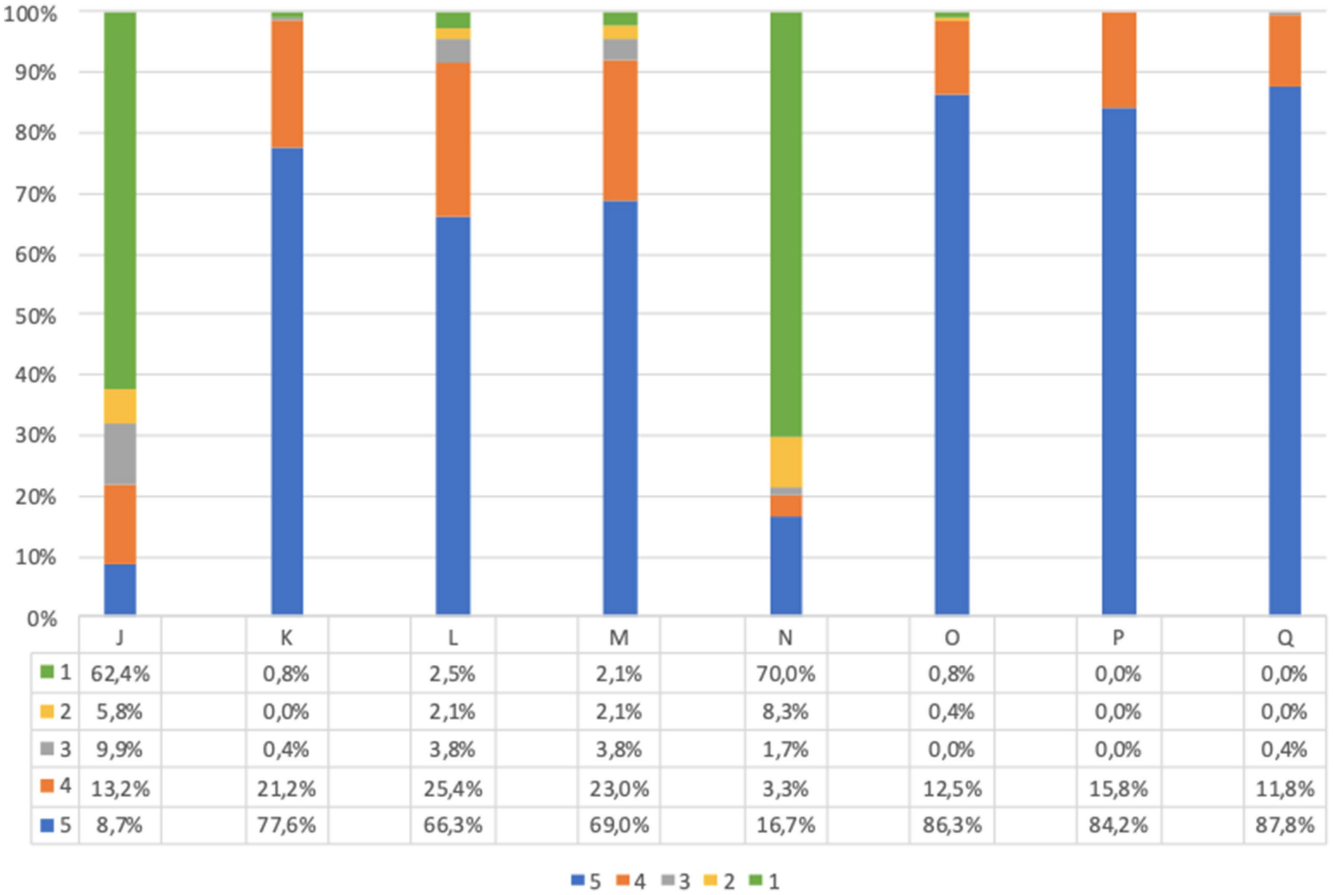
Children’s questionnaire feasibility answers (%) immediately before and immediately after screening procedure. Answers were given on a 5-point ordinal scale: strongly disagree (1); disagree (2); neutral (3); agree (4); and strongly agree (5). (J) I am concerned about the idea of my child taking a capillary sample. (K) If my child tests positive for type 1 diabetes and/or celiac disease, I would agree to make him/her take a confirmatory venous blood sample, as required by the program. (L) I believe capillary screening is practical and easy. (M) I would prefer capillary sampling for my child as a screening method over venous blood sampling. (N) What I expected from the program for my child was different from what he/she got. (O) I am satisfied with the service my child received. (P) All information about the program was clear and easy to understand. (Q) I would recommend this screening program. Missing data are available in Supplementary Tables S2, S3.

Before screening 9.7% of adults and 21.9% of children’s parents were worried about the idea of themselves or their children taking a capillary sample (question J, Figures 4, 5). However, after screening, 95.7% of adults and 91.7% of children’s parents asserted that capillary screening is practical and easy (question L, Figures 4, 5); furthermore, 87.5% of adult and 92.0% of parents for their children stated their preference on capillary blood sampling as a screening method over venous blood sampling (question M, Figures 4, 5).

**Figure 6:**
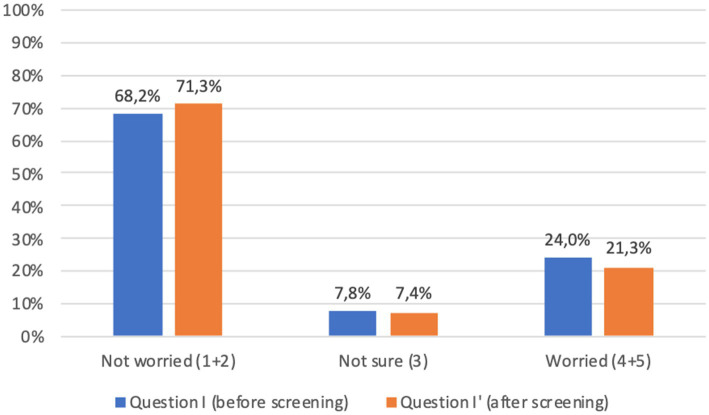
Adults answers to question I-I′ (%) immediately before and immediately after screening procedure: “I am concerned about the possibility of an early diagnosis or being identified as a person at risk.” Answers were given on a 5-point ordinal scale: strongly disagree (1); disagree (2); neutral (3); agree (4); and strongly agree (5). Missing data are available in Supplementary Tables S2, S3.

**Figure 7:**
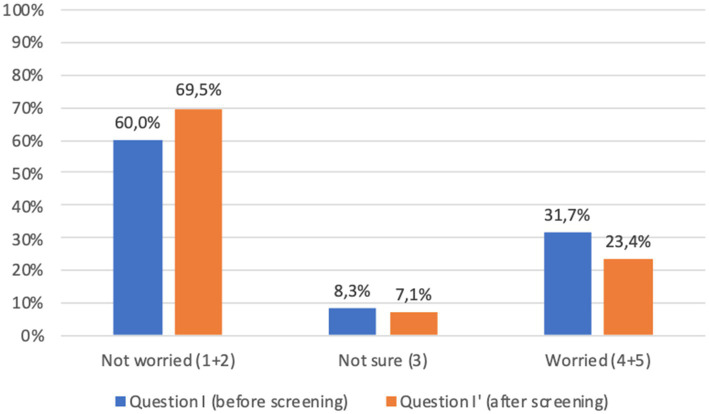
Children’s parents answers to question I-I′ (%) immediately before and immediately after screening procedure: “I am concerned about the possibility of my child receiving an early diagnosis or being identified as a person at risk.” Answers were given on a 5-point ordinal scale: strongly disagree (1); disagree (2); neutral (3); agree (4); and strongly agree (5). Missing data are available in Supplementary Tables S2, S3.

With regard to the psychological impact of screening (question I), before screening 24.0% of adults and 31.7% of children’s parents were worried about the idea of being diagnosed with a chronic autoimmune or metabolic disease; worries decreased after screening (question I′) to 21.3 and 23.4%, respectively. Comparison of the answers showed a significant difference (*p* < 0.001) between responses before and after screening, which was confirmed when considering separately adults and children’s parents (Figures 6, 7).

Lastly, the great majority of participants expressed overall satisfaction with the program (questions N to Q, Figures 4, 5).

### 3.4 Confirmatory venous sampling

Out of 81 participants candidate to retesting due to positivity for at least one autoantibody, confirmatory venous sampling was obtained in 69 participants (85.2%). Twelve of them (14.8%) were not re-tested.

Among these, only 4 (4.9% of those candidate to retesting, 0.3% of the total cohort) may be considered actual dropouts. The remaining 8 either performed confirmatory samples on their own, or had been previously diagnosed with diabetes or celiac disease.

### 3.5 Adverse events

Mild adverse events were reported in 38 participants (2.5%): 34 presented a vagal reaction/ lipothymia (one associated with vomiting), while 4 experienced anxiety/panic attacks. No serious adverse events were reported.

## 4 Discussion

The aim of the UNISCREEN Study was to assess the feasibility and acceptability of a population screening for chronic autoimmune, metabolic and cardiovascular diseases conducted universally, with no restrictions to participants, except for residency, including all age groups and using capillary blood sampling for disease risk markers assessment. The findings of the study show that this approach is feasible and acceptable.

To our knowledge, this is the first study introducing blood capillary screening as a procedure to be used across all age groups. Large studies, such as Fr1da (8–10), ASK (11, 12) and DiaUnion TRIAD (13), are conducted using capillary blood sampling for autoantibody screening for type 1 diabetes, celiac disease and other associated autoimmune diseases. However, these studies are focused on children and adolescents only. Moreover, none of these studies included metabolic markers besides antibodies. UNISCREEN is therefore unique, as the same blood capillary sample is used for screening not only autoantibodies, but also metabolic and cardiovascular disease markers, such as glucose, glycated hemoglobin and the lipid panel. For instance, with regard to lipids, only a few studies have used capillary blood for screening, such as Fr1dolin for LDL-hypercholesterolemia in very young children (14).

Feasibility and acceptability were primary objectives within the ambition of UNISCREEN, and both have been fulfilled as reported in the present study. Feasibility is supported by a number of results: first, recruitment for screening was above the pre-specified threshold of 50% (2) of the general population. Participation was not uniform across ages, with the highest response (almost 59%) obtained in the age group 1–15 years and the lowest in the age groups 16–30 and 75–100 (41.3 and 42.8%, respectively). This reflects on one side the effectiveness of awareness-raising campaign realized within UNISCREEN in nursery and primary schools, thanks to the active role of teachers; on the other hand it suggests insufficient efficacy of awareness campaigns in secondary schools and communities attended by teenagers and young adults. For young people there is a need to better employ the Internet and social media, the most widely used information channels in that age range (15). The response rate was also lower in the older adult age groups, most likely due to difficulties encountered by older adult people to move outside home to reach the screening point facility and may be also by an insufficient sensitization to the screening program. In order to improve the response rate in the older adult ages, screening with capillary sampling at home should be encouraged (16), as well as a deeper involvement of the local general practitioner, as suggested by several studies (17, 18).

A second pillar of feasibility was the practicability of blood capillary sampling and measurement. In 47.1% of cases, one single fingerprick was sufficient for collecting enough blood for extemporary measurement of all metabolic markers and for subsequent autoantibody assays; in 39.8% a second sample was needed, meaning that in 86.9% of cases the number of capillary samples required to perform the entire screening procedure was equal to or less than two. Moreover, capillary blood drawing was also efficient, as indicated by successful extemporary measurement of all the six metabolic markers concomitantly by POC devices in above 90% of participants and nearly 100% yielding for late autoantibody testing. These performances are remarkable, but possibly unique to UNISCREEN as a clinical trial, operating with a skilled staff of physicians and nurses responsible for capillary blood drawing, some with specific expertise with children. A transfer of this procedure to standard clinical practice would need confirmation. Feasibility evaluation using the questionnaire also indicated that more than 90% of participants felt that capillary sampling was an easy and painless procedure, with the vast majority expressing their preference for capillary over venous sampling, similarly to other studies (16, 19). Along the same line, adverse events were infrequent (2.5%) and of minor entity, mainly represented by anxiety/vaso-vagal reactions.

The questionnaires showed that the program was well received by participants, being clear, transparent and perceived as safe. Patient satisfaction is an increasingly important measure in health care, since studies have shown positive associations between overall patient satisfaction and clinical outcomes (20). This was also confirmed by the high response rate when confirmatory testing was required, with venous blood sampling occurring in a second occasion for those who were found positive to antibody screening, showing less than 5% of participants lost to follow-up.

A large percentage of participants shared the overall goal of screening, being convinced of its usefulness in improving public health and prevention: this matter is crucial, as population adherence is influenced by shared goals. This awareness increased after the screening procedure and the interview with the medical staff, as did the understanding of the diagnostic-therapeutic procedure to be followed in the event of a positive test result. Thus, a brief but effective medical consultation to review test results at the time of the screening process has shown to be key in enabling the procedure with a positive and concrete impact on people’s lives and their health.

The possible psychological impact of screening, particularly for chronic diseases such as type 1 diabetes in children (21), has been a source of debate in the scientific literature (22), and fear of being diagnosed may be one of the factors influencing lower adherence to screening programs. A significant proportion of participants in our study were indeed concerned about the possibility of receiving a diagnosis. However, this proportion decreased after screening, which may be related to the immediacy of some of the results, allowing to at least partially reduce the anticipatory anxiety. In addition, the medical consultation, with real-time interpretation of the results and concrete indications on treatment and follow-up, permitted to respond right away to many doubts and concerns of the participants.

Lastly, a total of 5.3% of participants tested positive for either type 1 diabetes or celiac disease autoantibodies on screening. This is an overall prevalence higher than expected based on data from the literature. However, the threshold for positivity of autoantibody measurement was deliberately chosen to be low enough to maximize sensitivity. It is likely that the majority, but not all, of those re-tested will confirm positivity.

Strengths of the study include the multi-comprehensive approach of the screening tests, both in terms of number of diseases screened and age groups included. The involvement of skilled and trained medical, nursing, as well as non-medical staff, is another strength of our study, along with the possibility of receiving part of the results in real time, providing immediate feedback and thereby increasing compliance and reducing the risk of losing participants to follow-up.

There is also an important limitation to our study: questionnaires were administered to people who voluntarily participated to the screening, which inevitably introduces some biases in response.

Our study was carried out in a small community, offering encouraging results regarding the feasibility of the project.

Translation of the UNISCREEN experience on feasibility to larger scale programs, such as dictated in Italy by the national law on screening for type 1 diabetes and celiac disease in the pediatric general population (23), is challenging. The proper training of dedicated personnel, the availability of adequate equipment, besides to a number of cultural, legal, political, and economic factors will need to be addressed. On the other hand, the introduction of a screening program by law, with the systematic involvement of GPs and family pediatricians, is a key instrument for the success of such an ambitious program.

Nonetheless, UNISCREEN represents the only example of general population screening study recently performed in Italy.

## 5 Conclusion

Our study demonstrates how a multi-comprehensive screening approach encompassing all ages for the most common metabolic and autoimmune diseases by capillary sampling is feasible and well accepted. Overall, these data further support the opportunity of using capillary blood testing in large-scale screening campaigns, as it could potentially improve adherence to prevention programs in the general population. This result is encouraging as it paves the way for larger studies at national level and beyond, setting a benchmark for future public health screening efforts.

## Data Availability

The raw data supporting the conclusions of this article will be made available by the authors, without undue reservation.

## UNISCREEN Study Group Collaborators

Sara Angiulli, Aurora Merolla, Alberto Bongiovanni, Rebecca De Lorenzo, Elena Rela, Rita Peracino, Chiara Bellino, Giuseppina Manganaro, Elisa Borgonovo, Sara Critelli, Aldo Fici, Martina Mallus, Giulia Morocutti, Giulia Pata, Clara Soggetti, Chiara Calabrese, Alberico Di Resta, Maria Pia Ruggiero, Antonella Spano’, Francesca Biasco, Manuel Stocchi, Pietro Melodia, Giuseppe Ancona, Andrea Laurenzi.

## References

[1] Wilson J Junger G. Principles and practice of screening for disease. World Health Organization; (1968). Available at: https://apps.who.int/iris/handle/10665/37650 (Accessed February 3, 2025).

[2] Merolla A De Lorenzo R Ferrannini G Renzi C Ulivi F Bazzigaluppi E. Universal screening for early detection of chronic autoimmune, metabolic and cardiovascular diseases in the general population using capillary blood (UNISCREEN): low-risk interventional, single-Centre, pilot study protocol. BMJ Open. (2024) 14:e078983. doi: 10.1136/bmjopen-2023-078983, PMID: 38448070 PMC10916121

[3] Wyatt RC Grace SL Brigatti C Marzinotto I Gillard BT Shoemark DK. Improved specificity of glutamate decarboxylase 65 autoantibody measurement using luciferase-based immunoprecipitation system assays. Diabetes. (2024) 73:565–71. doi: 10.2337/db23-0550, PMID: 38232306 PMC10958581

[4] Liberati D Wyatt RC Brigatti C Marzinotto I Ferrari M Bazzigaluppi E. A novel LIPS assay for insulin autoantibodies. Acta Diabetol. (2018) 55:263–70. doi: 10.1007/s00592-017-1082-y, PMID: 29305766

[5] Williams CL Marzinotto I Brigatti C Gillespie KM BOX Study Group Lampasona V. A novel, high-performance, low-volume, rapid luciferase immunoprecipitation system (LIPS) assay to detect autoantibodies to zinc transporter 8. Clin Exp Immunol. (2024) 215:215–24. doi: 10.1093/cei/uxad139, PMID: 38150393 PMC10876106

[6] Lampasona V Liberati D. Islet Autoantibodies. Curr Diab Rep. (2016) 16:53. doi: 10.1007/s11892-016-0738-2, PMID: 27112957

[7] Burbelo PD Lebovitz EE Bren KE Bayat A Paviol S Wenzlau JM. Extrapancreatic autoantibody profiles in type I diabetes. PLoS One. (2012) 7:e45216. doi: 10.1371/journal.pone.0045216, PMID: 23028856 PMC3448600

[8] Kick K Hoffmann VS Lange K Lang M Laub O Bechtold-Dalla Pozza S. Feasibility and organization of a population-based screening for pre-symptomatic type 1 diabetes in children—evaluation of the Fr1da study. J Public Health. (2019) 27:553–60. doi: 10.1007/s10389-018-0981-x

[9] Raab J Haupt F Scholz M Matzke C Warncke K Lange K. Capillary blood islet autoantibody screening for identifying pre-type 1 diabetes in the general population: design and initial results of the Fr1da study. BMJ Open. (2016) 6:e011144. doi: 10.1136/bmjopen-2016-011144, PMID: 27194320 PMC4874167

[10] Ziegler A-G Kick K Bonifacio E Haupt F Hippich M Dunstheimer D. Yield of a public health screening of children for islet autoantibodies in Bavaria, Germany. JAMA. (2020) 323:339–51. doi: 10.1001/jama.2019.21565, PMID: 31990315 PMC6990943

[11] Jia X He L Miao D Zhang C Rewers M Yu L. A high-throughput multiplexed screening for type 1 diabetes, celiac diseases, and COVID-19. JoVE. (2022) 185:e63787. doi: 10.3791/63787, PMID: 35876559 PMC10040254

[12] Stahl MG Geno Rasmussen C Dong F Waugh K Norris JM Baxter J. Mass screening for celiac disease: the autoimmunity screening for kids study. J Am College Gastroenterol ACG. (2021) 116:180–7. doi: 10.14309/ajg.0000000000000751, PMID: 32701732 PMC7775339

[13] Scherman MN Lind A Hamdan S Lundgren M Svensson J Pociot F. Home capillary sampling and screening for type 1 diabetes, celiac disease, and autoimmune thyroid disease in a Swedish general pediatric population: the TRIAD study. Front. Pediatrics. (2024) 12:1386513. doi: 10.3389/fped.2024.1386513, PMID: 38699153 PMC11063237

[14] Kordonouri O Lange K Boettcher I Christoph J Marquardt E Tombois C. New approach for detection of LDL-hypercholesterolemia in the pediatric population: the Fr1dolin-trial in Lower Saxony, Germany. Atherosclerosis. (2019) 280:85–91. doi: 10.1016/j.atherosclerosis.2018.11.011, PMID: 30496984

[15] Park E Kwon M. Health-related internet use by children and adolescents: systematic review. J Med Internet Res. (2018) 20:e120. doi: 10.2196/jmir.7731, PMID: 29615385 PMC5904452

[16] Liu Y Rafkin LE Matheson D Henderson C Boulware D Besser REJ. Use of self-collected capillary blood samples for islet autoantibody screening in relatives: a feasibility and acceptability study. Diabet Med. (2017) 34:934–7. doi: 10.1111/dme.13338, PMID: 28226181 PMC5816681

[17] Genovese C. Adherence to the three Italian screening in a sample of women (and men) in the southern Italy. Clin Ter. (2021) 172:75–9. 10.7417/CT.2021.2287 33346333

[18] Studts CR Tarasenko YN Schoenberg NE. Barriers to cervical cancer screening among middle-aged and older rural Appalachian women. J Community Health. (2013) 38:500–12. doi: 10.1007/s10900-012-9639-8, PMID: 23179390 PMC3600402

[19] Bingley PJ Rafkin LE Matheson D Steck AK Yu L Henderson C. Use of dried capillary blood sampling for islet autoantibody screening in relatives: a feasibility study. Diabetes Technol Ther. (2015) 17:867–71. doi: 10.1089/dia.2015.0133, PMID: 26375197 PMC4677115

[20] Glickman SW Boulding W Manary M Staelin R Roe MT Wolosin RJ. Patient satisfaction and its relationship with clinical quality and inpatient mortality in acute myocardial infarction. Circ Cardiovasc Qual Outcomes. (2010) 3:188–95. doi: 10.1161/CIRCOUTCOMES.109.900597, PMID: 20179265

[21] O’Donnell HK Rasmussen CG Dong F Simmons KM Steck AK Frohnert BI. Anxiety and risk perception in parents of children identified by population screening as high risk for type 1 diabetes. Diabetes Care. (2023) 46:2155–61. doi: 10.2337/dc23-0350, PMID: 37673098 PMC13158050

[22] Johnson SB. Psychological impact of screening and prediction in type 1 diabetes. Curr Diab Rep. (2011) 11:454–9. doi: 10.1007/s11892-011-0208-9, PMID: 21710195

[23] Bosi E Catassi C. Screening type 1 diabetes and celiac disease by law. Lancet Diabetes Endocrinol. (2024) 12:12–4. doi: 10.1016/S2213-8587(23)00354-6, PMID: 38048797

