# Supplementary material for: Universal capillary screening for chronic autoimmune, metabolic and cardiovascular diseases: feasibility and acceptability of the UNISCREEN study
Source: Front Public Health. 2025 Feb 11;13:1506240. doi: 10.3389/fpubh.2025.1506240 (PMC11850345; doi:10.3389/fpubh.2025.1506240)
Supplement: Supplementary file 1 [file Data_Sheet_1.docx]

Supplementary Material

# Supplementary Tables

## Supplementary Table 1

| Feasibility and Acceptability questionnaire |
| --- |
| Before screening |
| A) I believe this screening program will be useful for general/paediatric population to prevent future health problems. |
| B) I believe this screening program will be useful for me/my child to prevent future health problems. |
| C) I support the idea of population screening for diabetes, celiac disease and cardiovascular disease. |
| D) I believe this program will improve my/my child's quality of life. |
| E) I believe this program will help me/my child change my/his/her lifestyle. |
| F) I find this program safe in terms of health effects and data confidentiality. |
| G) I have understood what I/my child would have to do in case of a positive screening test result. |
| H) I understood the purpose of the program. |
| I) I am concerned about the possibility of an early diagnosis or me/my child being identified as a person at risk. |
| J) I am concerned about the idea of me/my child taking a capillary sample. |
| K) If I/my child test positive for type 1 diabetes and/or coeliac disease, I would agree to take a confirmatory venous blood sample, as required by the program. |
|  |
| After screening |
| A’) I believe this screening program will be useful for general/paediatric population to prevent future health problems. |
| B’) I believe this screening program will be useful for me/my child to prevent future health problems. |
| C’) I support the idea of population screening for diabetes, celiac disease and cardiovascular disease. |
| D’) I believe this program will improve my /my child's quality of life. |
| E’) I believe this program will help me/my child change my lifestyle. |
| F’) I find this program safe in terms of health effects and data confidentiality. |
| G’) I understood what I/my child would have to do in case of a positive screening test result. |
| H’) I understood the purpose of the program. |
| I’) I am concerned about the possibility of an early diagnosis or me/my child being identified as a person at risk. |
| L) I believe capillary screening is practical and easy. |
| M) I would prefer capillary sampling as a screening method over venous blood sampling for me/my child. |
| N) What I expected from the program was different from what I/my child got. |
| O) I am satisfied with the service I/my child received. |
| P) All information about the program was clear and easy to understand. |
| Q) I would recommend this screening program. |

**Supplementary Table 1.** Feasibility and Acceptability questionnaire. Question A to K were administered to the participant immediately before screening; question A’ to Q immediately after screening. For participants under 16 years of age, answers were given by a parent.

## Supplementary Table 2

| Missing data of baseline characteristics (N=1535) | |
| --- | --- |
| Smoking, n(%) | 28 (1.8%) |
| Family setting, n(%) | 30 (2.0%) |
| BMI, n(%) | 30 (2.0%) |
| Lifestyle, n(%) | 34 (2.2%) |
| Pregnancy, n(%) | 20 (1.3%) |
| Gestational diabetes, n(%) | 20 (1.3%) |
| Diabetes Mellitus, n(%) | 29 (1.9%) |
| Hypertension, n(%) | 29 (1.9%) |
| Cardiovascular events, n(%) | 50 (3.3%) |
| Active therapy for Diabetes Mellitus, n(%) | 35 (2.3%) |
| Active therapy for hypertension, n(%) | 29 (1.9%) |
| Active therapy for dyslipidaemia , n(%) | 29 (1.9%) |
| Cardioaspirin, n(%) | 32 (2.1%) |
| Oral contraceptives, n(%) | 16 (1.0%) |
| Number of fingerpricks, n(%) | 9 (0.6%) |

**Supplementary Table 2.** Missing data of baseline characteristics of screened population of

Cantalupo.

## Supplementary Table 3

| Missing data of answers to "Feasibility and Acceptability questionnaire" (N=1535) | |
| --- | --- |
| Question A, n(%) | 5 (0.33%) |
| Question B, n(%) | 4 (0.26%) |
| Question C, n(%) | 5 (0.33%) |
| Question D, n(%) | 6 (0.39%) |
| Question E, n(%) | 5 (0.33%) |
| Question D, n(%) | 9 (0.59%) |
| Question G, n(%) | 11 (0.72%) |
| Question H, n(%) | 7 (0.46%) |
| Question I, n(%) | 6 (0.39%) |
| Question J, n(%) | 9 (0.59%) |
| Question K, n(%) | 9 (0.59%) |
| Quesion A', n(%) | 10 (0.65%) |
| Question B', n(%) | 11 (0.72%) |
| Question C', n(%) | 14 (0.91%) |
| Question D', n(%) | 11 (0.72%) |
| Question E', n(%) | 11 (0.72%) |
| Question F', n(%) | 13 (0.85%) |
| Question G', n(%) | 16 (1.04%) |
| Question H', n(%) | 11 (0.72%) |
| Question I', n(%) | 14 (0.91%) |
| Question L, n(%) | 11 (0.72%) |
| Question M, n(%) | 12 (0.78%) |
| Quesion N, n(%) | 17 (1.11%) |
| Question O, n(%) | 19 (1.24%) |
| Question P, n(%) | 11 (0.72%) |
| Question Q, n(%) | 16 (1.04%) |

**Supplementary Table 3.** Missing data of answers to “Feasibility and Acceptability questionnaire”.

# Supplementary Figures

## Supplementary Figure 1

**Supplementary Figure 1:** Gender response rates (%) in different age groups.

## Supplementary Figure 2

**Supplementary Figure 2.** Number of fingerpricks performed during screening to complete all measurements (%). Missing data, n(%)=11(0.7%).

### 2.2 Supplementary Figure 3

**Supplementary Figure 3**. Number of fingerpricks (1 or more than one) in different age groups (%). Missing data, n(%)=11(0.7%).
